# The Relative Importance of Various Subjects in Practical Sanitation

**Published:** 1882-02

**Authors:** 


### POPULAR SCIENCE DEPARTMENT.

### THE RELATIVE IMPORTANCE OF VARIOUS SUBJECTS IN PRACTICAL SANITATION.

DR. C. B. WHITE'S PRESIDENTIAL ADDRESS BEFORE THE AMERICAN PUBLIC HEALTH ASSOCIATION, AT SAVANNAH, GA., NOV. 29, 1881.

Members of the American Public Health Association, I welcome you to this ninth annual meeting.

On the 12th of September, 1872, twelve gentlemen met at Long Branch, elected to membership other three present by invitation, and held the first meeting, that of organization of this Association.

To one then present, it is pleasant to note that its members number seven hundred, and that, aided by a fortunate combination of circumstances, nearly four hundred members were in attendance at the meeting at New Orleans. Of those present at that meeting of organization, which we now see was so important and opportune, two are dead—Carl Pfeifer, Esq., Civil Engineer and Architect, and Dr. John M. Woodworth, late Surgeon General of the United States Marine Hospital Service. Had they done no other worthy work, their presence, and interest, and practical part in forming this Association, would be monumental.

It is my official and sad duty to announce the death during the past year of Dr. George S. Blackie, Nashville, Tenn.; Dr. Greensville Dowell, Galveston, Texas; Dr. W. C. W. Glazier, United States Marine Hospital Service; Dr. E. Lloyd Howard, Baltimore, Md.; Dr. L. S. Joynes, Richmond, Va.; Dr. Charles H. Smith, Richmond, Va.; Dr. E. M. Wight, Chattanooga, Tenn.

The time allotted to this address is much too short to justly set forth the many and valuable services of these dead associates. I therefore recommend to the Association the creation of a committee, which, before we separate, may place before us the leading events of their lives, and, prepare a memorial for the volume of the year's transactions, which shall fully illustrate their services and virtues.

Physicists tell us that the warmth, light, and life of our earth depend on rapidly recurring, incessantly repeated oscillations, propagated through space by the mysterious, inconceivably great energies of the sun. Every brief undulatory wave of force has its value, duly performs its work, surpassed by neither successor or predecessor.

Every generation of man has its own work, labor impossible to be done by one antecedent, or a following one. Neither has the work of the one going before, or of that coming after, greater importance. The generation or the man thus does the best possible, has done as well as those before him have done, or as successors can do. The curse of the world is its idlers awaiting opportunity.

The man who does the thing next him that requires doing, has no need to wait for occasions—he makes opportunity.

Endeavor to benefit by the consolations of philosphy as we may, all thoughtful persons can but greatly lament the loss of men of fine intellectual powers, matured by labor, experience and time, dying at such period of life, that to benefit mankind there might be justly expected ten or twenty years of clearest thought, of well directed, practical work of the best quality, of wise and wide influence.

These, our associates, have ceased to labor, some of them while comparatively young, feeling that they had learned a little, only enough to begin to study to advantage, and were eagerly looking forward to more rapidly gained increments of knowledge and to riper and more abundant accomplishments in the future. As with them so with others; the plow may be stopped afield in the furrow. For some of us the time is assuredly short. Let us take our lesson of self-sacrifice and industry and honesty of purpose, from these our dead, and turn to the living, to our duties and opportunities, from the contemplation of what they worthily did, to what there is for us to do.

Early in the year a circular of inquiry was sent to the members of the Advisory and Executive Committee, and to former Presidents of the Association, requesting them to name those topics of sanitary science which seemed to them worthy of especial consideration at the Savannah meeting. In the replies forty different subjects were so characterized. Of those mentioned by more than one person, were five; disinfectants recommended, by two; the parasites, or diseases of food animals, by three; house hygiene, by three; vital statistics, nomenclature, registration, etc., by three; hygiene of the school and scholar, by four.

The most peculiar replies from our "sanitary philosophers" were these:

"The time of the Association could be best occupied in correcting the false theories announced at former meetings." Another suggests for discussion "whether there be such a thing as sanitary science."

We may construe the lessons of these numerous and so varied answers to be, that those interested in the science of hygiene and pushing its advance along a well-extended front, that much of our work is done on the principle of a man's doing what he finds to do "over against his house."

That some theories have been adventurously thrown out, much as some railways have been advanced on cribwork laid upon the long-grassed sod of Louisiana's "trembling prairies," across which travelers get by audacity, velocity and luck, but which the steady, slow-going, scarcely noticed construction trains of a later date will fill in, be it with solid material or accumulated fact, till the shaking roadway and the swift, but fortunate transit, becomes the established, the sure, the safe.

Others of theories advanced to serve as highways of science, are, I greatly fear, as the Slough of Despond, wherein Christian was so woefully bemired, in which Bunyan tells us had been swallowed up at least twenty thousand cart loads, yea! millions of wholesome instructions, but it remained still a slough.

The idea that there is no such thing as sanitary science, even though jocosely suggested, or as an occasion of wit in others, to create discussion, would not be surprising if seriously meant.

Almost the whole of the enormous outgrowth of modern discovery and classified knowledge belongs to the present century, and it may be said of hygiene as of the doctrine of evolution, that it has barely come of age. Sanitary science is a dependence and consequence of general scientific research and progress.

Observers have long existed. We have had men prescient far in advance of the generations, sanitarians, vital statisticians laboring to-day, the sure foundations of preventive medicine.

But a glance to-day at the condition of even the records of mortality throughout the United States shows a lack almost disheartening of accurate statistics. A few cities and towns, with perhaps two or three States, have a fully recorded mortality.

The registration of diseases, even of those contagious, is, with possibly a few exceptions, only a well meaning and well deserving attempt.

To-day our most honest, philosophical minds, those whose opportunities for forming correct judgment have been longest and best, are in doubt whether yellow fever be a disease always imported, or from former importation now become endemic, or occasionally imported and sometimes surviving mild winters, or be an indigene in some way bred from malaria or evoked from filth, or be always home-bred and in-bred, a glandular disease, perhaps, developed by general bad environments. Such contrariety of opinion could assuredly have been merged into the unanimity of knowledge had there been twenty years of observations, conducted with scientific accuracy and completeness.

Scientific methods and the scientific quality of mind are foreign to many of those who are interested students, and even writers on hygiene. The scientific order of mind can be bred as we develop certain qualities in our domestic animals. As scientific studies are made more familiar to scholars, and scientific modes of thought and teaching are applied in the education of the young, and increasingly regulate the habit of thought of later years, so generation upon generation will become better suited by hereditary, innate quality of intellect to seek and obtain truth by exact methods. As we are almost at the beginning of the knowledge of proper methods, so we are only at the beginning of the proper use of them, and can hopefully look forward to the appearance of steadily increasing numbers of those vastly better furnished intellectually to apply and use them than is the present generation. In the immediate future of all science progress promises to be "progression," perhaps even "geometrical."

Many statements assumed to be facts, numerous, hasty, generalizations, based upon incomplete, unfair, or imperfectly recorded observations, theories eagerly thrust forward by illy-constituted or badly trained minds, or truths perverted or misused to fill a pocket, or build a "balloon frame" reputation, appear in the scientific columns of newspapers, or occasionally find places in journals devoted to genuine research and to the discussion of well founded working theories. Soon refuted by the logic of events, or the later theory of another pseudo-scientist, or cut through by the merciless edge of fact in the hand of one of the elect of truth, theory and theorists disappear together.

The resonant, glittering, but empty brass, no longer blinds the eye or stuns the ear. Unfortunately, others similarly plausible or impudent, follow in so numerous or extended procession as to cause those not so well founded in underlying principles to say: "Is there such a thing as sanitary science?" The old perplexity cries out afresh: "What is truth?"

Brief consideration of certain qualities of mind necessary to the perfect man of science, instead of causing discouragement at slow progress, will rather cause surprise at the advance made.

To avoid self-deception is the most difficult of problems. Like the perfected balance of the chemist and physicist, shut out from all perturbing influences, affected by and accurately recording the most minute accretion, the perfectly ordered scientific mind, free even of personal equation, would perceive and yield to, and appreciate the smallest increment of fact, no matter to which end of the beam it lay. In apparent antithesis, such a mind would be, not merely receptive, but hospitable to foreign or novel ideas.

Far from neutral, it would be eager for knowledge, penetrated with a very hunger for truth. Therefore we may happily be encouraged, seeing that whilst man is imperfect, and his capacities limited and immature, so much has been accomplished by a sincere desire of truth, and an earnest purpose to lessen the miseries, and increase the enjoyments of mankind.

In periodicals and papers, and from the lecture stand, we hear of filth and "filth diseases." We are told that "to be clean is next to being godly," for as the one insures the salvation of the soul, so the other saves the body. Every outbreak of disease is announced as due to neglect of some form of washing. Next, a scientific commission reports that the workmen and their families, employed and living for years at some "knackery" in the midst of decaying and corrupted animal matters, breathing an atmosphere loaded with animal particles, and of a stench unbearable to those not made tolerant by long exposure to it, nevertheless enjoy good and continuous health.

All medical men have observed children born and brought up in unwholesome localities, with eminently unsavory surronndings scarcely washed between birth and marriage, yet unmistakably in possession of firm health.

I have in mind a man whose domicile is in a swampy location, and whose premises as a whole have been a standing nuisance for thirty years, offensive to the nostrils and eyes of neighbors, and for whose exceeding bad condition he has been subjected to repeated suits by the health authorities. This man lives in mud and manure, not over cleanly, and frequently drunken, his only water supply an above-ground uncovered wooden cistern, on whose margin chickens roost, having one sanitary virtue—but that one the greatest of them all—he is a good eater! Despite unfavorable environments and habits he has high health, quickly recovers from accidents, and unless untimely slain by one of them, bids fair at some future "All Saints' Day" to visit the graves of the sanitary inspectors and officers who have successively assaulted him.

The existence of such facts, or the announcement of such statements as facts, surprise and confound most readers.

(*To be Concluded.*)

